# Fabella Syndrome Following De-Rotation Surgery to Correct a Femoral Malunion

**DOI:** 10.2174/1874325001812010346

**Published:** 2018-08-29

**Authors:** Iain Rankin, Haroon Rehman, George Patrick Ashcroft

**Affiliations:** NHS Grampian, Aberdeen Royal Infirmary, Aberdeen, UK

**Keywords:** Fabella, Fabella Syndrome, Modified Surgical Technique, Arthroscopy, Case report, Knee

## Abstract

**Background:**

The fabella is a sesamoid bone situated within the lateral head of the gastrocnemius tendon, close to the lateral femoral condyle, and adjoined to the fabellofibular ligament. It is a normal variant, found in up to 87% of patients. Fabella Syndrome describes traditionally posterolateral knee pain, occurring due to biomechanical pressure of the fabella against the lateral femoral condyle. Given its rarity, its diagnosis is often overlooked. We present a case of Fabella Syndrome with a modified surgical excision technique and review of the literature.

**Methods and Results:**

A thirty-four-year-old man presented with posterolateral knee pain following de-rotation surgery to correct a femoral malunion, from a previous femoral shaft fracture. Due to the patient’s complex orthopaedic history, Fabella Syndrome was not initially diagnosed. Fabellectomy eliminated all symptoms of knee pain, with no limitations in knee function.

**Conclusion:**

Review of the literature identified ten publications (evidence level IV) describing Fabella Syndrome. This is the first reported case of Fabella Syndrome secondary to femoral de-rotation surgery. The authors recommend fabellectomy as a definitive treatment for Fabella Syndrome, in keeping with published literature.

## 1 INTRODUCTION

The fabella is a sesamoid bone situated within the lateral head of the gastrocnemius tendon, close to the lateral femoral condyle, and adjoined to the fabellofibular ligament. Its presence is a normal variant, with a prevalence reported as ranging from 20 to 87% [1-7]. It is situated within the lateral head of gastrocnemius tendon, with the fabellofibular ligament (or ligament of Vallois) connecting it to its distal insertion at the fibular head [8]. It is bordered posteriorly by the oblique popliteal ligament [2]. Anteriorly, it is bordered by the posterior capsule of the knee. Its location within the posterolateral aspect of the knee places it within intersecting lines of tensile stress and articulating with the lateral femoral condyle [5]

The presence of a fabella is usually asymptomatic. Fabella Syndrome refers to posterolateral knee pain associated with the presence of a fabella. The presence of pain occurs due to biomechanical pressure of the fabella against the lateral femoral condyle. It is characterized by periodic posterolateral pain which is exacerbated during extension, where tension causes the fabella to compress against the lateral femoral condyle [9]. On occasion, its presence can result in irritation of the common peroneal nerve, causing neurologic symptoms of numbness or dysesthesia [10].

The presence of a fabella may be palpated during clinical examination. Lateral radiographs of the knee can confirm the presence of a fabella. CT and MRI imaging can delineate the position of a fabella in relation to the posterolateral femoral condyle. Ultrasound has been described as a useful adjunct to provide information regarding the posterolateral structures of the knee and their relation to a fabella [11, 12].

Fabella syndrome has been treated conservatively with manual therapy involving extension, flexion and rotation of the knee [13]. Where conservative management has failed, operative treatment may be performed. Surgical techniques described include open, arthroscopic, and arthroscopic assisted open excision [8, 14, 15].

## 2 CASE REPORT

A 34-year-old gentleman was first referred to orthopaedic services with a history of persistent knee pain, located over the right femoral condyle near the origin of the lateral collateral ligament. Eleven years prior to presentation, the patient was involved in a road traffic accident where he sustained a fracture of the right femoral neck and ipsilateral shaft. Initial management consisted of open reduction, fixation of the femoral neck fracture with cannulated screws and the ipsilateral shaft fracture with plating. The patient later had a revision of the plate to a femoral nail. Union was subsequently achieved with the femoral shaft fracture; however, a significant external rotation deformity was noted, and discomfort to the knee.

The patient was referred on to our tertiary referral orthopaedic centre for femoral de-rotation surgery. An initial CT scan performed revealed an external rotation deformity of 45 degrees. The patient procedure included removal of femoral nail, osteotomy with de-rotation surgery, and subsequent exchange nail. The femoral nail was fixed proximally and locked into place distally, following the corrective 45 degrees of internal rotation achieved to the distal femur during osteotomy, performed under intraoperative radiographic guidance. The post-operative rehabilitation regime consisted of initial touch weight bearing only, with gradual increments in weight bearing status. At six months follow-up, the patient complained of distal lateral femur pain. A repeat CT scan was performed, revealing a delayed femoral union. This was initially thought to be the cause of the patient’s symptoms. The patient was admitted for dynamization of the femoral nail with an injection of bone graft substitute, and iliac crest graft. Despite eventual union, the patient continued to complain of pain at the distal lateral femur, with a cracking sensation on movement now noted.

**Image (1):**
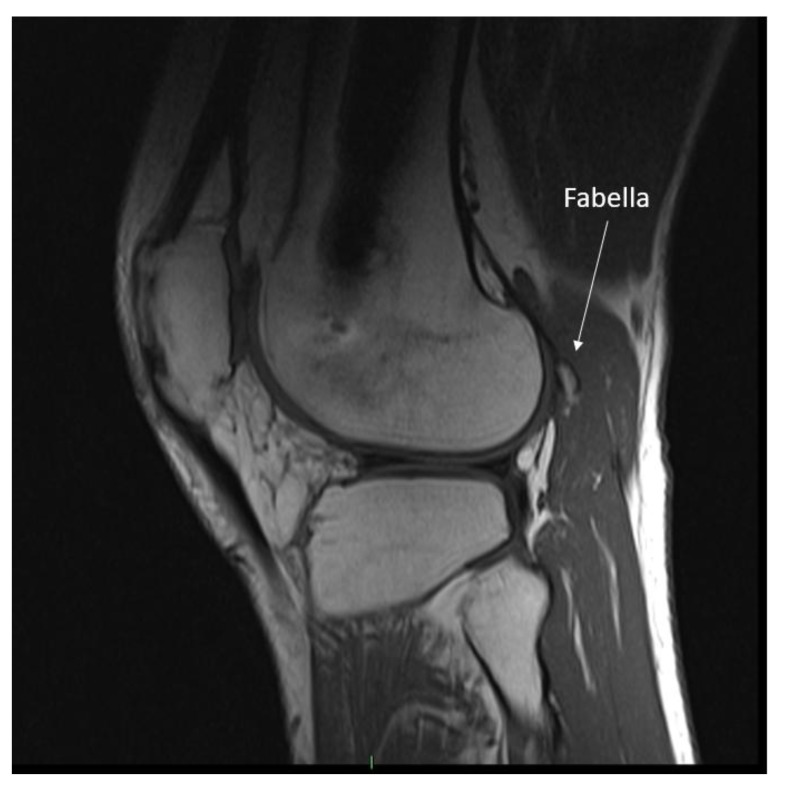
MRI revealing fabella embedded within the lateral head of gastrocnemius.

**Image (2):**
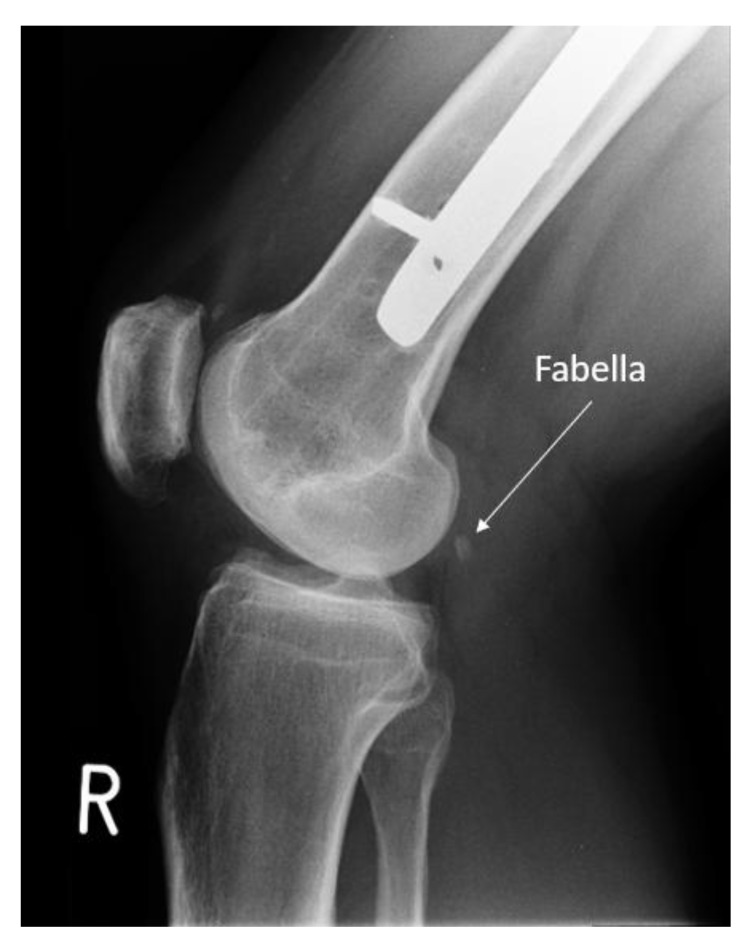
Review of sagittal radiographs revealing fabella.

**Image (3):**
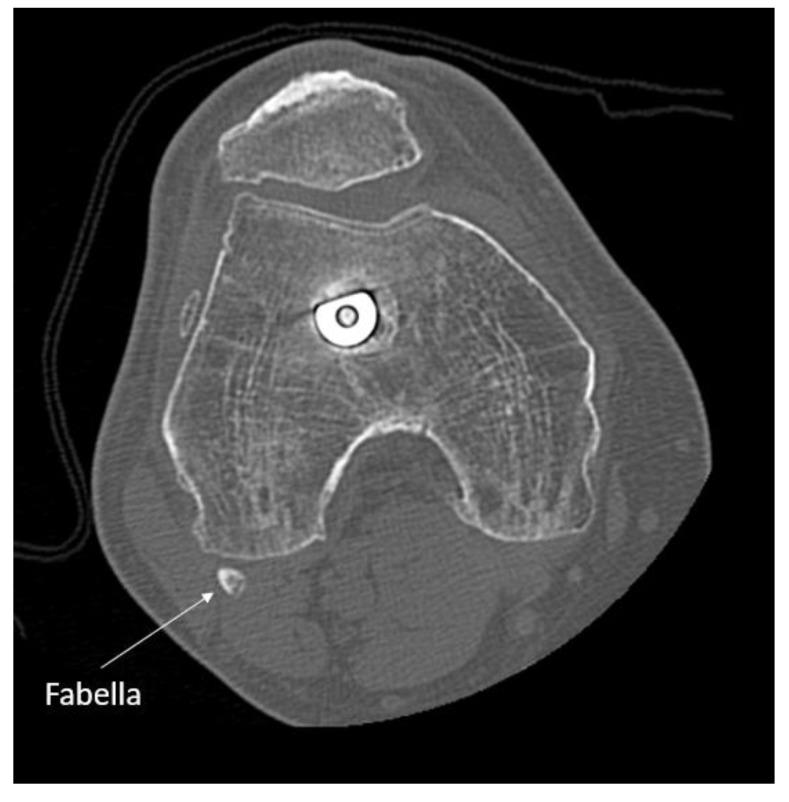
Review of coronal CT revealing fabella.

Repeat clinical examination revealed a palpable crepitus over the distal iliotibial band with a snapping sensation, as it appeared to catch. A palpable small, solid swelling was noted at the posterolateral right knee. Ultrasound and CT revealed intra-articular loose bodies lying within the lateral para-patellar gutter. The patient subsequently underwent arthroscopy with removal of loose bodies. Despite this, at six-months follow-up to arthroscopy, the patient continued to complain of posterolateral knee pain. MRI showed no evidence of abnormality to the popliteus tendon or muscle, but highlighted the fabella embedded within the lateral head of gastrocnemius at the posterolateral corner of the knee. (Image **1**). The fabella was also notable on radiographs (Image **2**) and CT (Image **3**). Ultrasound scan confirmed the fabella as mobile on movement and associated with tenderness. The diagnosis of Fabella Syndrome was made, and the patient planned for a Fabellectomy procedure.

**Image (4):**
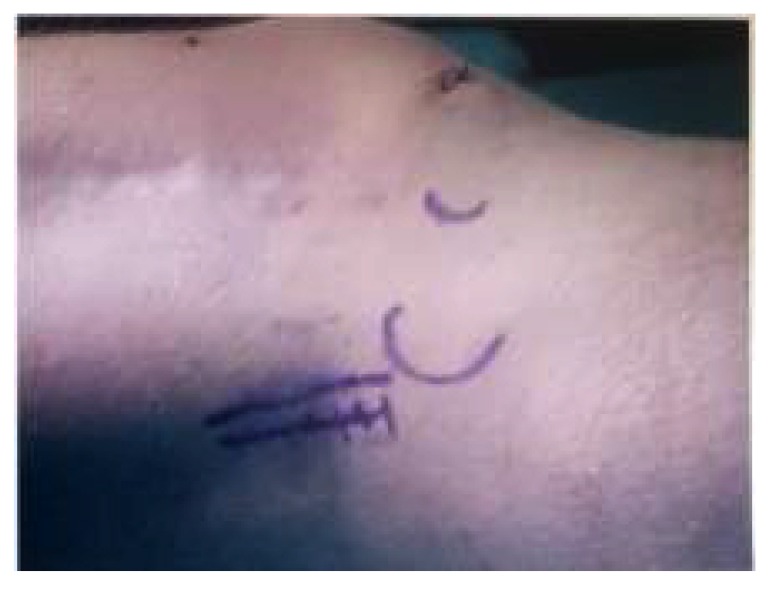
Minimally invasive approach used directly over palpable fabella. Most posterior line utilised. Also marked are Gerdy’s tubercle and the head of the fibula.

**Image (5):**
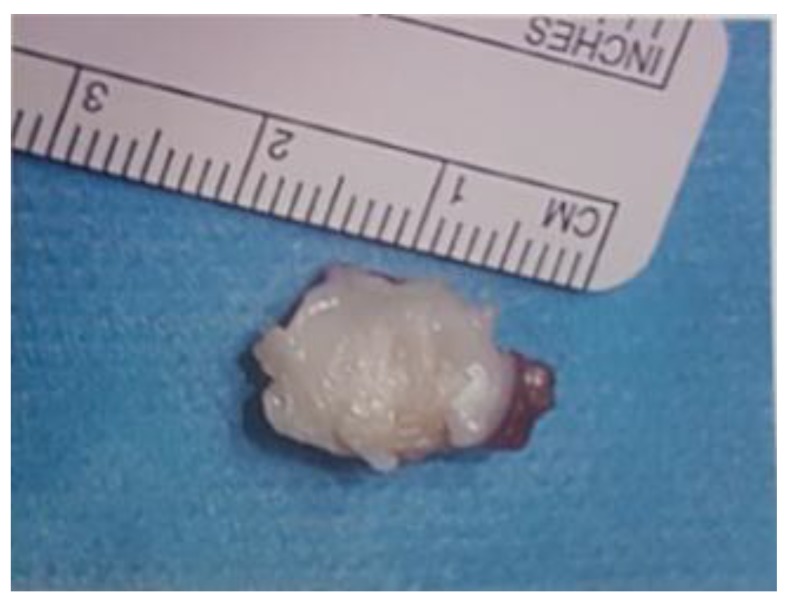
The excised fabella.

An initial arthroscopy was performed to review the knee joint, and to potentially assist in surgical excision of the fabella. A complete diagnostic arthroscopy was performed, with the fabella unable to be identified through the posterior capsule. After arthroscopy, open fabellectomy was performed. In view of the patient’s extensive scarring from prior orthopaedic procedures, a short 2cm incision was made directly over the palpable fabella (Image **4**). The common peroneal nerve was identified and spared. Subsequent incision left a cuff of biceps femoris to protect the nerve. Gastrocnemius was split and incised directly over the fabella, which was removed in entirety (Image **5**). Histopathology confirmed the diagnosis.

The patient was followed-up at two and ten months postoperatively. At both follow-ups, he described the complete resolution of his posterolateral knee pain.

## 3 DISCUSSION

Fabella syndrome is traditionally described as a cause of pain in the posterolateral aspect of the knee. A literature review revealed ten publications describing fabella syndrome [8, 9, 13, 14, 16-20]. All publications were evidence level IV, with six case reports and four case series, the largest of which described 16 patients [9]. Across each publication, posterior knee pain was present in all patients. Other described features include posterolateral knee swelling, pain on direct pressure of the fabella, pain on extension of the knee, clicking sensations, and common peroneal nerve dysfunction. Fabella syndrome has been described secondary to total knee arthroplasty [16, 21-25], however, we describe the only known documented case of fabella syndrome occurring secondary to femoral de-rotation surgery.

The diagnosis of fabella syndrome is not always immediately clear, particularly in the presence of concurrent orthopaedic pathology - as highlighted in this case. Despite its innocuous appearance and rarity, the diagnosis should not be overlooked. A detailed history should include the presence of posterolateral knee pain, with the possibility of other symptoms as described above. Standard clinical examination must include palpation of the posterolateral structures of the knee. Reproducible pain upon applying pressure to the fabella suggests fabella syndrome as the underlying cause. Other pathologies including osteochondral fragments, foreign bodies, Baker’s cyst, meniscal tears, and localized pigmented villonodular synovitis, should be excluded prior to diagnosis. Standard anteroposterior and lateral radiographs of the knee should be utilised to reveal most bony fabella, with MRI utilised to aid in the diagnosis of smaller or cartilaginous fabellae. A Japanese cadaveric study found 66% of subjects to have the presence of a fabella - 29.3% of subjects had an entirely bony fabella, with the remainder displaying osteochondral or cartilaginous fabella [4].

In most of the reported cases, immediate and persistent pain relief was produced with fabellectomy. Conservative treatment with physiotherapy was found to be successful in a minority of cases. Zipple *et al* describe in detail the technique of manual mobilisation of the fabella to achieve pain relief [13]. The largest case series described five out of sixteen patients (31%) responding to conservative treatment. The remaining eleven patients required surgery – all of which obtained immediate and persistent relief of symptoms [9].

Surgical excision has been described arthroscopically, arthroscopically assisted and directly via an open technique. Dannawi *et al* described arthroscopic excision of the fabella in two patients [14]. Provencher *et al* describe an arthroscopic assisted fabella excision, with initial arthroscopy to help delineate the borders of the fabella, prior to open excision [15]. The remaining fabellectomy procedures described within the literature review utilised an open technique. As per previous authors, an arthroscopic evaluation of the knee prior to excision of the fabella was performed to aid in delineating the borders of the fabella, which was not possible in this case. Subsequently, open excision was performed. In keeping with published literature, fabellectomy provided immediate and persistent pain relief.

No cases of Fabella Syndrome secondary to femoral de-rotation surgery were identified within the published literature. The patient, in this case, required a significant femoral correction (45 degrees) with consequent alteration in the relative tensions of the surrounding soft tissues in the post-operative period. The resultant Fabella Syndrome was thought to be caused secondary to the increased tensile stress of the fabella upon the lateral femoral condyle, as in keeping with other causes of Fabella Syndrome [5]. Increased soft tissue tension of the fabellofibular ligament, oblique popliteal ligament, and the lateral head of gastrocnemius tendon were all hypothesised to contribute towards an increase in the tensile stress of the Fabella articulating with the lateral femoral condyle and resultant Fabella Syndrome.

An alternative diagnosis within the differential of this patient following de-rotation surgery is that of common peroneal nerve irritation [10]. The common peroneal nerve may have been stretched following de-rotation surgery, to correct a 45-degree femoral malunion in this patient. A differential in the diagnosis could include common peroneal nerve irritation subsequent to this. As fabellectomy provided total resolution of the patient’s symptoms, with sparing of the peroneal nerve (which was protected intraoperatively), the diagnosis of Fabella Syndrome was made.

## CONCLUSION

This paper describes the first reported case of Fabella Syndrome secondary to femoral de-rotation surgery. In keeping with the published literature of Fabella Syndrome secondary to other causes, fabellectomy provided total resolution of symptoms. The authors recommend fabellectomy as a definitive treatment for Fabella Syndrome secondary to femoral de-rotation surgery.
